# Paravalvular Leak After Transcatheter Aortic Valve Replacement (TAVR): A Literature Review

**DOI:** 10.3390/jcm14248905

**Published:** 2025-12-16

**Authors:** Giorgio Sciaramenti, Edoardo Menzato, Stefano Clo’, Carmen Izzo, Laura Rotondo, Beatrice Dal Passo, Sofia Meossi, Renè Tezze, Federica Frascaro, Elisabetta Tonet, Federico Marchini, Marta Cocco, Carlo Tumscitz, Carlo Penzo, Gianluca Campo, Rita Pavasini

**Affiliations:** Cardiology Unit, Azienda Ospedaliero-Universitaria di Ferrara, 44124 Ferrara, Italy; giorgio.sciaramenti@gmail.com (G.S.); edoardo.menzato@gmail.com (E.M.); stefanoclo7@gmail.com (S.C.); carmen.izzo2017@gmail.com (C.I.); laura.rotondo11@gmail.com (L.R.); beatrice.dalpasso@edu.unife.it (B.D.P.); sofimeossi@gmail.com (S.M.); rene.tezze@gmail.com (R.T.); federica.frascaro92@gmail.com (F.F.); tonet.elisabetta@gmail.com (E.T.); federico.marchini@unife.it (F.M.); mcocco92@gmail.com (M.C.); tumscitz@gmail.com (C.T.); carlopenzo78@gmail.com (C.P.); cmpglc@unife.it (G.C.)

**Keywords:** TAVR, aortic regurgitation, leak, paravalvular leak

## Abstract

Severe aortic stenosis represents a significant prognostic burden, particularly in symptomatic patients. The advent of transcatheter aortic valve replacement (TAVR) has revolutionized the treatment of patients previously considered ineligible for surgical aortic valve replacement (SAVR). TAVR provides a relatively safe intervention that leads to improvements in survival, symptoms, and functional status within months of implantation. A major complication following TAVR is the occurrence of paravalvular leaks (PVLs), which have been associated with increased mortality and higher rates of heart failure-related hospitalizations. PVLs refer to abnormal blood flow between the implanted valve and the aortic wall, which can compromise the functionality of the device. Careful pre-procedural planning enables the identification of patients at higher risk for PVL development. Although the incidence of PVLs has decreased with the introduction of newer-generation transcatheter valves, the condition remains clinically relevant. Due to the complex anatomy of the aortic valve apparatus and interference from the prosthetic frame, accurate evaluation of PVLs requires a multimodal diagnostic approach. Current evidence on PVL management is limited. In most cases, a conservative approach is adopted, while interventional strategies (such as pre- and post-dilatation, percutaneous PVL closure, and TAVR-in-TAVR) are reserved for selected patients. We performed a systematic literature review to summarize the incidence, predictors, diagnostic techniques, and management strategies of PVLs following TAVR.

## 1. Introduction

Transcatheter aortic valve replacement (TAVR) has become the standard of care for patients with severe aortic stenosis who are over 70 years of age with favorable anatomy (tricuspid aortic valve, permissive transfemoral access, porcelain aorta) and/or classified at high or prohibitive predicted surgical risk [1].

From a procedural perspective, TAVR has experienced global growth, with increasing numbers of procedures performed worldwide and the accumulation of experience among centers and operators. The recent European Guidelines for the Management of Valvular Heart Disease have lowered the age threshold for considering TAVR from 75 to 70 years, reflecting the growing feasibility of the procedure, the expanding range of available bioprosthetic valve models, and the continuous refinement of implantation techniques [1]. The contemporary TAVR landscape is dominated by two valve platforms: (i) balloon-expandable valves (BEV), characterized by cobalt–chromium frames, bovine pericardial leaflets, and high radial force; and (ii) self-expanding valves (SEV), nitinol frames with supra-annular leaflet design. This type of bioprosthesis, once released from the delivery system, expands itself to its programmed shape exerting a continuous radial force on the aortic annulus [1]. Both platforms employ balanced radial expansion mechanisms and rely on anchoring to the surrounding anatomical structures, supported by sealing skirts designed to fill residual gaps between the prosthesis and the native annulus, thereby reducing the risk of paravalvular leak [1].

Although TAVR was initially developed as the only option for patients at high surgical risk, it has been recently validated for patients at intermediate and low surgical risk [1]. The PARTNER III trial, demonstrated that in 1000 low-risk patients, TAVR with the Sapien 3 valve resulted in non-inferior outcomes compared to surgical aortic valve replacement (SAVR), with no significant differences in the 1-year composite primary endpoint of all-cause mortality, disabling stroke, non-disabling stroke, or rehospitalization and in the 5-year secondary endpoints including all-cause death or disabling stroke, new-onset atrial fibrillation, aortic-valve reintervention, endocarditis, and clinically significant valve thrombosis [2].

Despite these advancements, paravalvular leak (PVL) remains one of the most frequent complications following TAVR. Data from the PARTNER trials and other large registries indicate that 50–85% of patients experience some degree of PVL post-TAVR. While the majority of leaks are trivial or mild, moderate to severe PVL is observed in approximately 5–10% of cases, depending on valve type, size, and implantation technique [2,3].

Accurate quantification and clinical assessment of PVL are essential, as moderate or severe leaks have been associated with worse long-term outcomes, including increased mortality. Consequently, understanding the mechanisms, diagnosis, and management strategies of PVL is critical for improving post-procedural outcomes.

The aim of this review is to provide a comprehensive overview of PVL in the context of contemporary TAVR practice. We discuss the incidence of PVL across different transcatheter valve platforms—namely self-expandable and balloon-expandable prostheses—and explore current diagnostic modalities, staging criteria, and available treatment options based on the latest evidence and evolving device technologies.

## 2. Risk Factors for Paravalvular Leak (PVL)

Unlike surgical aortic valve replacement, transcatheter aortic valve replacement does not involve excision of the native valve. Instead, the prosthetic device is implanted within the native valve, displacing the leaflets outward toward the aortic root. This implantation method can create gaps between the prosthetic stent frame and the native aortic annulus, resulting in paravalvular leak, a complication significantly more frequent after TAVR than after SAVR [4].

### 2.1. PVL Incidence in TAVR Versus SAVR

In 2020, Makkar et al. published a subanalysis of the PARTNER II trial evaluating five-year outcomes of TAVR using a balloon-expandable bioprosthesis (Sapien XT, Edwards Lifesciences, Irvine, CA, USA) compared to SAVR in patients with severe aortic stenosis at intermediate surgical risk, defined as an estimated 30-day surgical mortality between 4% and 8% according to the STS risk score [5]. Conducted across 57 centers in the United States and Canada and enrolling 2030 patients, the study demonstrated no significant differences between TAVR and SAVR in terms of the composite primary endpoint of all-cause mortality or disabling stroke at five years.

Despite similar valve hemodynamic performance between the two groups, the incidence of PVL was significantly higher in the TAVR cohort compared to the SAVR cohort (33.3% vs. 6.3%, respectively). The presence of PVL was therefore proposed as the primary hypotheses to explain the observed difference in late outcomes—particularly mortality and disabling stroke—considering that, when PVLs are moderate to severe, they have has been associated with adverse prognostic implications.

The SURTAVI trial, published in 2022, also addressed the comparison between SAVR and TAVR in patients with severe aortic stenosis and intermediate surgical risk, as defined by STS-PROM criteria [6]. In this trial, patients in the TAVR arm received either a first-generation (CoreValve, Medtronic, Dublin, Ireland) or second-generation (Evolut R, Medtronic) self-expandable bioprosthesis, while patients in the SAVR arm underwent surgical valve implantation with the choice of prosthesis left to the treating physician (excluding mechanical valves).

With respect to PVL, the trial confirmed that, although TAVR patients had lower mean transvalvular gradients and larger effective orifice areas, the incidence of PVL remained higher compared to the surgical cohort. Mild PVL occurred in 27.1% of TAVR patients (*n* = 98) versus 2.7% in SAVR patients (*n* = 8). Moderate or greater PVL was reported in 3.0% (*n* = 11) of the TAVR group and 0.7% (*n* = 2) of the SAVR group, corresponding to a risk difference of 2.37% (95% CI, 0.17–4.85%; *p* = 0.05).

### 2.2. Anatomical Risk Factors

The most significant anatomical predictor of PVL is valve calcification, which can be quantitatively assessed using the Agatston calcium score. In early-generation devices, a score >3000 was strongly associated with at least moderate PVL [7]. In later generations, sex-specific thresholds have been identified, with scores >4070 in men and >2341 in women independently predicting moderate PVL [8].

The role of calcification in TAVR is extremely important, as it can be one of the most critical factors in the possible complication of paravalvular leak. Unlike SAVR, where surgical removal of calcium allows the new prosthesis to be positioned without difficulty, TAVR involves autonomous expansion of the bioprosthesis, which can be limited by eccentric or bulky calcifications, making this one of the major predictors of PVL [8].

**Figure 1:**
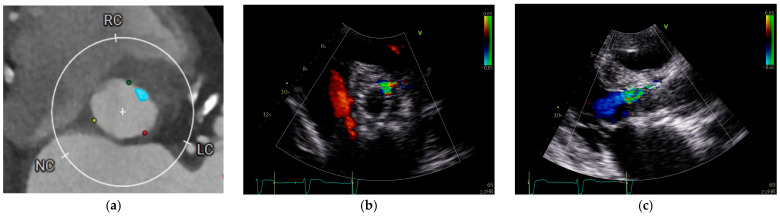
Annular calcification and residual PVL after TAVR. (**a**) Cardiac computed tomography performed for pre-TAVR planning revealed an annular calcification located at the level of virtual basal ring between the left and right coronary cusps. (**b**,**c**) Following TAVR, an anterior paravalvular leak persisted at the site of the previously documented calcification, approximately at the 1 o’clock position.

However, not only the quantity but also the distribution of calcification plays a key role. The presence of calcifications in the leaflets, annulus, and left ventricular outflow tract (LVOT), as well as asymmetric patterns of deposition, have all been independently associated with increased PVL risk [9,10,11]. The eccentricity index, calculated as [1 − (Dmin/Dmax)], where Dmin and Dmax represent the smallest and largest orthogonal diameters of the aortic annulus as measured by multislice computed tomography (MSCT), is another important predictor when >0.25 [12]. Moreover, bicuspid aortic valve morphology and extreme annular dimensions (either small or large) further contribute to higher PVL incidence. Other anatomical risk factors include discrepancy between LVOT and annulus diameter (leading to poor prosthesis apposition) and a steep aorto-LVOT angulation, which may alter radial deployment forces and stent geometry [13,14,15]. Table 1 presents the principal risk factors for PVLs. Figure 1 shows an example of correlation between pre-implantation annular calcification and residual PVL after TAVR.

### 2.3. Procedural Risk Factors

**Figure 2:**
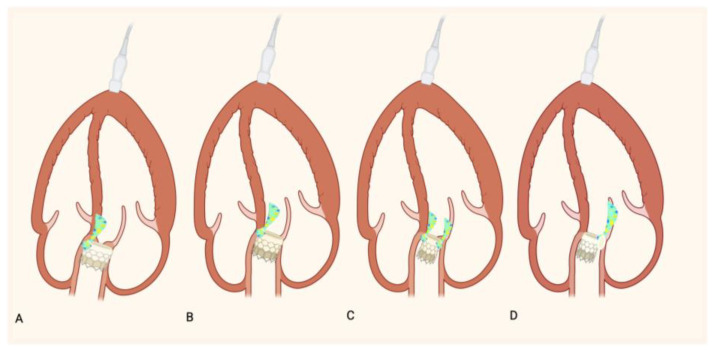
Incorrect prosthesis positioning: (**A**) Schematic representation of an excessively shallow (**B**) and deep implant and PVL. Schematic representation of a hypo- expansion of TAVR without (**C**) and with (**D**) extensive calcification and PVL.

Technical factors during TAVR implantation can also significantly influence PVL risk. Incorrect prosthesis positioning—either too high or too deep—can lead to poor sealing. Similarly, hypo expansion of the prosthesis may occur due to insufficient radial force or extensive calcification, increasing the likelihood of PVL [14,16,17]. Figure 2 summarizes these aspects.

The type of valve and mode of deployment are also associated with PVL outcomes. Balloon-expandable (BE) valves exert greater radial force at deployment, which may facilitate better sealing compared to self-expandable (SE) valves, particularly in cases of heavy or asymmetric calcification [15].

### 2.4. Prosthesis Sizing and Cover Index

A crucial consideration during TAVR planning is avoiding prosthesis–annulus mismatch, a known risk factor for PVL. One tool to guide prosthesis sizing is the cover index, calculated as:

Cover Index = (Prosthesis diameter − Annulus diameter)/Prosthesis diameter) × 100. A low Cover Index predicts a major risk of PVLs [14]. Historically, annular measurements were obtained using transesophageal echocardiography (TEE), but MSCT has become the gold standard. MSCT not only provides more accurate and typically larger annular diameters compared to TEE but also allows assessment of annular eccentricity, enabling more precise prosthesis selection [15,18]. The use of MSCT has thus led to improved prosthesis sizing, reducing the risk of PVL.

The recent availability of intermediate valve sizes has further optimized prosthesis–annulus matching, especially in anatomies falling between traditional sizing intervals, thus contributing to the overall decline in PVL incidence.

### 2.5. PVL Incidence in Balloon Expandable Verus Self-Expandable Bioprosthesis

A key step in the management of patients referred for TAVR is meticulous pre-procedural planning, which relies primarily on cardiac computed tomography (CCT). CCT offers high spatial resolution, enabling precise assessment of aortic root and valvular anatomy. Over time, the potential of CT imaging in this setting has become increasingly evident, and the lack of structured pre-procedural assessment during the early TAVR experience likely contributed to the initially high rates of paravalvular leak (PVL) observed with first-generation prostheses. Transcatheter heart valves (THVs) can be broadly categorized into two main types based on their deployment mechanism: self-expandable and balloon-expandable prostheses. SE valves (e.g., Evolut PRO/PRO+, corevalve—Medtronic, Allegra THV—New Valve Technology, Navitor TAVI System—Abbott) utilize a frame composed of shape-memory alloys such as Nitinol, which allows the device to expand automatically upon release. BE valves (e.g., SAPIEN 3 Ultra—Edwards Lifesciences, Myval—Meril Life Sciences, Vapi, Gujarat, India), on the other hand, are deployed through the inflation of a balloon mounted on the delivery catheter, which forces the metallic stent frame to expand at the annular level.

The OPERA-TAVI registry represents one of the most robust comparative analyses evaluating the incidence of PVL based on prosthesis type [19]. This multicenter study included 2241 patients undergoing TAVR with either the SE Evolut PRO/PRO+ or the BE SAPIEN 3 Ultra valves. Results demonstrated comparable rates of moderate to severe PVL between the two groups; however, mild PVL was more frequent in the SE valve cohort. Self-expandable valves were associated with a higher incidence of PVL, largely due to their lower immediate radial force and greater susceptibility to eccentric annular calcification, which can impair uniform frame apposition compared with the more predictable and forceful expansion achieved with balloon-expandable valves. In the OPERA-TAVI registry, despite similar device-success rates between self-expanding (SE) and balloon-expandable (BE) valves (SE: 87.4% vs. BE: 85.9%; *p* = 0.47), the SE group exhibited higher rates of conduction disturbances (permanent pacemaker implantation 17.9% vs. 10.1%; *p* < 0.01) and disabling stroke (2.3% vs. 0.7%; *p* = 0.03). Self-expanding nitinol frames exert a progressive yet more prolonged radial force in the subannular region, precisely where the His bundle and left bundle branch course, leading to sustained compression of these structures. In addition, these devices typically require a deeper implantation within the left ventricular outflow tract, further increasing the likelihood of conduction disturbances. Secondly, incomplete expansion of the self-expanding valve could contribute to creating asymmetries in the subannular frame, resulting in abnormal pressure in certain sensitive areas of the interventricular septum, which could contribute to the development of conduction disorders [19].

These findings confirm earlier observations from the SOLVE-TAVI trial [20] and reinforce the notion that patient-specific anatomical factors—such as annular size, calcification burden, and distribution—may have a more significant impact on PVL development than the specific prosthesis model itself.

Another notable trial is the CHOICE trial, a randomized controlled study comparing the SE corevalve (Medtronic) and the BE SAPIEN XT (Edwards) in approximately 240 high-risk patients with severe aortic stenosis [21]. At 1-year follow-up, a higher incidence of moderate PVL was observed in the SE group. However, by 5 years, the number of patients with at least moderate PVL had decreased by half, with only 6 of the initial 12 cases persisting. This suggests a potential regression of PVL over time, though the authors also emphasized the challenges of standardized PVL assessment and the limitations of long-term echocardiographic follow-up in this frail population. Of note, among the 12 patients with moderate PVL at 1 year, 3 had died and 3 lacked follow-up imaging data at 5 years.

While awaiting results from the SMART trial (Small Annuli Randomized to Evolut or SAPIEN), expected in 2028, Okuno et al. conducted a five-year echocardiographic follow-up study using propensity score matching to compare SE (corevalve Evolut) and BE (SAPIEN) valves in patients with severe aortic stenosis and small annuli (defined as annular area <430 mm^2^) [22]. Although SE valves demonstrated more favorable hemodynamic parameters (lower gradients, larger effective orifice area), these did not translate into improved clinical outcomes at five years. Moreover, moderate or greater PVL remained more prevalent in the SE group. This finding supports the conclusion that, in patients with small annuli, BE valves may be associated with a lower risk of PVL, despite some debate on the clinical significance of mild PVL. In the study by Okuno et al., balloon-expandable valves were less prone to PVL because their immediate high radial force and more uniform annular expansion provided a more predictable seal than the gradual, anatomy-dependent expansion of self-expandable device. In addition, many modern BE have optimized sealing skirts, which help to seal any small irregularities and reduce residual PVL [23].

In contrast, evidence for large annuli remains limited. Correct assessment of annular dimensions is critical in TAVR planning, as an inadequate cover index is associated with increased PVL risk. The ACURATE neo2 XL valve (Boston Scientific, Marlborough, MA, USA), a newer-generation SE prosthesis with larger diameters, has shown promising early results. In a cohort of 13 patients with CT-derived annular diameters between 26.5 and 29 mm, Gooley et al. reported no deaths or strokes at 30-day follow-up, and 92% of patients had no or only trace/mild PVL. However, the small sample size warrants caution in interpreting these results [24,25,26,27]. Unfortunately, in May 2025, following the outcomes of the ACURATE IDE trial, in which the valve did not meet the predetermined non-inferiority margin for safety and effectiveness compared to other devices, Boston Scientific discontinued the commercial distribution of the ACURATE valve system. Several factors may account for the outcomes observed in this trial. A primary limitation was the limited operator experience and the absence of a fully standardized procedural approach. In contrast to other self-expanding transcatheter valves, such as Sapien 3 or Evolut FX, the ACURATE system frequently required pre-dilation to prevent under-expansion of the bioprosthesis—a phenomenon reported in nearly 20% of procedures. Under-expansion was associated with suboptimal leaflet coaptation, which in turn contributed to a higher incidence of adverse events, including thromboembolic complications (i.e., myocardial infarction and stroke) and early structural valve deterioration. Moreover, unlike other contemporary THVs, this device did not permit complete full resheathing and recapture during deployment, thereby reducing procedural flexibility [28].

The Myval valve (Meril Life Sciences), a BE prosthesis, offers an expanded range of valve sizes with 1.5 mm incremental diameters, compared to the conventional 3 mm steps in other this. In the LANDMARK trial, moderate to severe PVL occurred in 3% of patients receiving Myval versus 5% in those implanted with other contemporary devices, with a relative risk reduction of 1.1% (*p* = 0.58). Although not statistically significant, this trend—combined with a lower post-dilatation rate (10% for Myval vs. 21% for other THVs)—suggests that custom sizing may help reduce PVL and other procedural complications, such as annular rupture or conduction disturbances [29].

Although no longer available today, a specific mention must be made of the Lotus valve (Boston Scientific), a mechanically expandable bioprosthesis distinct from both SE and BE devices. The Lotus valve incorporates a unique delivery and deployment system that enables repositioning after full deployment with a controlled mechanical expansion system which allows precise final positioning. Its polyurethane adaptive seal and sealing skirts were pioneering in minimizing PVL. In a secondary analysis of the REPRISE III trial, we have the comparison between the Lotus valve vs. SE corevalve/Evolut R in 912 high-risk patients across 55 centers [30]. At 5 years, mild PVL was significantly lower in the Lotus group (0.8% vs. 23.1%, *p* = 0.006), while moderate to severe PVL rates were similar between groups (0% vs. 1.9%, *p* = 0.31). However, this valve is now obsolete and no longer in clinical use, although its unique anchoring system was pioneering and served as a foundation for the design of subsequent bioprosthetic valve models. The technical reasons for withdrawing the valve from the market were primarily related to the large-profile introducer sheaths, which increased the incidence of vascular complications; the high radial force, which effectively minimized PVL but resulted in a higher risk of conduction disturbances; and the overall rigidity of the delivery system, which limited maneuverability and reduced adaptability in tortuous anatomies.

### 2.6. Other Clinical Predictors

Beyond anatomical and procedural variables, peripheral arterial disease and low body mass index (BMI) have also been identified as independent predictors of PVL in multiple studies [15,16,17], potentially reflecting more advanced vascular disease or technical challenges during valve deployment. Finally, TAVR in patients with bicuspid aortic valve (BAV) disease represents an area of growing clinical interest. However, despite its increasing use, the current literature lacks dedicated, large-scale studies evaluating the efficacy and safety of newer-generation transcatheter bioprosthesis in this specific anatomical setting. Notably, with the exception of the NOTION 2 trial, most randomized studies comparing TAVR and SAVR have systematically excluded patients with BAV [31].

A recent nationwide observational study conducted in Sweden addressed the comparative performance of SE versus BE valves in the BAV population [32]. The study included patients who underwent TAVR between 2016 and 2022, excluding those treated for aortic regurgitation or undergoing valve-in-valve procedures. The analysis revealed similar all-cause and periprocedural mortality rates between SE and BE prostheses. However, the incidence of more-than-mild PVL was significantly higher in the SE valve group (6.9%) compared to the BE group (1.5%) (adjusted odds ratio [aOR]: 7.47; 95% CI: 2.32–24.1; *p* < 0.001).

These findings align with prior literature, reinforcing the notion that SE valves are more prone to PVL than BE valves, particularly in anatomically complex settings such as BAV. Several pathophysiological mechanisms have been proposed to explain this difference. Firstly, asymmetric calcification—particularly in the non-coronary cusp—is more common in BAV and can hinder the complete expansion and sealing of SE valves, even after pre- or post-dilatation or oversizing strategies. Secondly, the elliptical geometry of the annulus, characteristic of BAV, may further limit the radial expansion of SE devices, which already exert lower radial force compared to BE prostheses. This may result in incomplete apposition of the valve to the annulus and the formation of residual gaps, predisposing the patient to PVL.

The term ‘radial force’ refers to the valve’s ability to expand during and after deployment at the native valve level, ensuring proper positioning by resting on the surrounding structures. Insufficient radial force could cause the valve to under-expand, increasing the likelihood of complications such as paravalvular leaks or thromboembolic events. Excessive radial force, on the other hand, can lead to complications such as annulus rupture or conduction disturbances [14,16,17].

Nevertheless, the use of a self-expandable valve in bicuspid aortic anatomy may be favored owing to its ability—particularly in the case of the Evolut valve—to accommodate an oval annular geometry. Conversely, balloon-expandable valves promote greater circularity but may increase the risk of annular rupture in the presence of heavy LVOT calcification.

Overall, while TAVR is increasingly feasible in selected BAV patients, these anatomical challenges underscore the importance of careful pre-procedural assessment and potentially favors the use of balloon-expandable valves in cases where PVL risk is a primary concern.

## 3. Multimodality Imaging for the Assessment of PVLs

Accurate evaluation of prosthetic valve dysfunction, particularly paravalvular leaks, requires a multimodal imaging approach. Standard diagnostic criteria for native valve disease are often inadequate due to the anatomical complexity of TAVR candidates and the diversity of prosthetic models. Moreover, discrepancies between imaging findings and clinical severity necessitate integrative multimodality assessment.

Angiography, typically performed immediately post-implantation, serves as the initial tool for detecting peri- or transvalvular regurgitation. However, echocardiography remains the primary modality for PVL detection, quantification, and longitudinal follow-up [33,34,35,36]. TEE is especially valuable in acute settings and procedural guidance, as well as in long-term surveillance and interventional planning. Additional imaging techniques include cardiac magnetic resonance (CMR) and ECG-gated computed tomography (CT), which complement echocardiographic findings. A complete overview of criteria for prosthetic aortic valve regurgitation is summarized in Table 2.

### 3.1. Angiographic and Hemodynamic Evaluation

Cine-angiography performed during valve deployment visualizes PVLs via iodinated contrast injection into the ascending aorta, with grading based on left ventricular opacification (Sellers classification, 1964) [37]. This method identifies four grades but is limited by its invasiveness, subjective interpretation, variability in catheter positioning, modest correlation with other modalities, and inability to differentiate central from paravalvular regurgitation [15,34]. Importantly, angiographic grading is not specifically validated for PVLs post-TAVR [34].

Quantitative densitometry of contrast time-density curves in the aortic root and left ventricular outflow tract offers a more objective alternative, demonstrating good correlation with echocardiography and CMR [38,39,40].

Hemodynamic indices, such as the Aortic Regurgitation (AR) Index, provide quantitative assessment by exploiting the relationship between regurgitation severity and pressure gradients [41]. It is defined as:

- AR index = [(DBP − LVEDP)/SBP × 100];
- DBP= diastolic blood pressure (mmHg);
- LVEDP= left ventricular end-diastolic pressure (mmHg);
- SBP= systolic blood pressure (mmHg).

An AR index <25% predicts increased 1-year mortality regardless of PVL severity [42]. Nonetheless, these invasive measures are affected by ventricular and aortic compliance, heart rate, and cannot distinguish PVL from intraprosthetic leaks [15,34].

Despite their advantages over angiography, hemodynamic assessments are limited by invasiveness and physiological confounders, restricting their utility in routine follow-up.

### 3.2. Echocardiographic and Color Doppler Assessment of Paravalvular Leaks

According to the American Society of Echocardiography (ASE) and the Valve Academic Research Consortium (VARC), echocardiography—particularly with Doppler—is the first-line imaging modality for the evaluation of prosthetic valve function and the identification and grading of paravalvular regurgitation.

However, PVL assessment presents specific challenges. Unlike native valve regurgitation, PVLs are often multiple, eccentric, and located at varying levels along the stent frame, usually confined to the LVOT. Image quality is frequently limited by heavy annular calcification and the prosthetic metallic frame, causing acoustic shadowing. Consequently, a comprehensive transthoracic echocardiography (TTE) study across multiple planes—including off-axis views—is essential, often requiring integration with TEE. TTE and TEE are complementary: TTE offers better alignment for LVOT flow quantification and superior assessment of left ventricular function, while TEE provides enhanced resolution for posterior PVLs [34,35,43].

Three-dimensional TEE adds anatomical detail but suffers from reduced temporal resolution, limiting sensitivity for small or transient jets [14].

While intravalvular regurgitation can generally be assessed using standard criteria, periprosthetic regurgitation often precludes full quantitative evaluation. Therefore, PVL grading largely relies on qualitative and semi-quantitative Doppler parameters [34,35,36].

#### 3.2.1. Structural and Morphological Evaluation

Morphological assessment may help elucidate underlying PVL mechanisms, such as:

- Prosthesis malpositioning (too high or low);
- Stent underexpansion or eccentricity;
- Extensive annular calcification;
- Visible gaps between the stent and native annulus.

Parasternal long- and short-axis views are optimal for this purpose. Three-dimensional imaging may aid in delineating structural abnormalities [34].

#### 3.2.2. Left Ventricular Size and Function

New-onset LV dilation post-TAVR may indicate significant regurgitation. However, in the acute phase, this is an unreliable marker. In chronic settings (≥3–6 months), moderate-to-severe PVLs are typically associated with LV enlargement. Stable LV dimensions during follow-up may suggest only mild PVLs [34].

#### 3.2.3. Color Doppler Assessment

Short-axis imaging across multiple levels of the prosthesis is essential, from leaflet base to the distal stent in the LVOT. For reproducibility, PVL jets are often mapped using a “clock-face” model, with the septal tricuspid leaflet at 9 o’clock. PVLs most commonly occur at 1–2, 5–6, and 9–11 o’clock—typically at commissural zones [9].

The circumferential extent of PVLs—expressed as a percentage of stent circumference—is a key semi-quantitative marker. Regurgitation is considered severe if >30%. However, assessment is complicated by jet multiplicity, eccentricity, and depth-dependent Doppler variation. Therefore, jet extent should be interpreted alongside additional Doppler and structural parameters.

#### 3.2.4. Scoring Systems and Reproducibility

Several scoring systems have been proposed to improve reproducibility:

- LAX Score: Evaluates six jet locations across PLAX, apical 3-, and 5-chamber views. Each location scores 1 point if regurgitation is present, 2 if a well significant jet is present, 0 if no jet neither turbulence is present; for echo-view anterior and posterior location has to be assesed (max score: 12). This is a purely qualitative score [44].
- CD Score: Combines the circumferential extent (CE%) from PSAX with the LAX score (CD = CE% + LAX). A score ≥30 suggests severe PVL.

A 2016 study of 165 patients showed only partial concordance between angiographic (Sellers) and echocardiographic grading. However, correlation improved when multiple echocardiographic parameters were combined—especially when using the LAX and CD scores. Notably, up to 17% of PVLs were missed in PSAX due to acoustic shadowing, highlighting the value of apical views and angiography in selected cases [44].

Interestingly, >60% of TAVR-related PVLs originate between 11 and 3 o’clock, near the right coronary sinus—differing from post-surgical PVLs, which more commonly originate between the right and non-coronary sinuses [44].

#### 3.2.5. Timing of Echocardiographic Follow-Up

Beyond intra-procedural imaging, the first post-TAVI echocardiographic assessment should be performed prior to hospital discharge. Subsequent follow-ups are recommended at 30 days and annually [45]. In cases of suspected dysfunction, imaging intervals should be tailored based on clinical symptoms, type of prosthesis, and nature of the abnormality.

### 3.3. Cardiac Magnetic Resonance in the Assessment of PVLs

CMR is the primary adjunct to echocardiography in the multimodality assessment of PVLs. It is considered the gold standard for quantification of biventricular volumes, mass, and function, and enables accurate and highly reproducible measurement of regurgitant volume (RV) and regurgitant fraction (RF), independent of jet number or morphology [34,35].

CMR is particularly valuable in cases of suboptimal echocardiographic windows, reclassifying PVL severity in over 40% of such patients [46]. The most validated technique is phase-contrast velocity mapping in the ascending aorta (short-axis, just above the prosthesis), which measures antegrade and retrograde flow to derive RV and RF [47]. Suggested RF cut-offs: <30% mild, 30–50% moderate, ≥50% severe PVL [45]. CMR’s advantages over echocardiography include operator independence, absence of acoustic limitations, and the ability to acquire images across unlimited planes [33]. Despite its accuracy, CMR has several limitations: (I) long acquisition times, with poor tolerance in claustrophobic or frail patients; (II) Motion artifacts due to arrhythmias or high heart rate; (III) Flow turbulence in PVLs may reduce image quality; (IV) Potential overestimation of RV due to diastolic coronary flow contamination, which may explain higher rates of severe PVLs reported by CMR compared to TEE (up to 2–3×) [34,48].

Alternative methods—such as calculating transvalvular regurgitant volume as the difference between LV stroke volume and pulmonary flow—are available but less precise [15].

CMR grading has demonstrated prognostic utility. In a study comparing discharge TEE with CMR performed ~40 days post-TAVR, CMR-derived RF >30% was the strongest predictor of 2-year mortality and heart failure hospitalization—mirroring findings in native aortic regurgitation [47].

Interestingly, regurgitant volume alone showed weaker correlation with outcomes, likely due to the small LV volumes and hypertrophic remodeling common in TAVI patients. In these patients, even a modest regurgitant volume may produce a high RF and exert significant haemodynamic burden, possibly explaining why mild PVLs have also been linked to adverse events [46]. Due to limited availability and high cost, CMR is not routinely used, but is recommended when: echocardiographic windows are suboptimal, there is discordance between imaging and clinical findings, further evaluation of ventricular function or remodeling is needed [15,34,46].

Newer protocols—such as 2D multi-venc sequences and 4D flow mapping—offer promising alternatives. These allow faster acquisitions (2D multi-venc) or free-breathing protocols (4D flow), improving feasibility in frail or multimorbid TAVR patients [33].

### 3.4. Cardiac CT in the Assessment of PVLs

Electrocardiogram-gated cardiac CT is primarily employed in the pre-procedural planning for TAVI. It enables precise anatomical assessment of the aortic root and surrounding structures—including annular dimensions, calcification burden and distribution, coronary ostia location, and the angulation between the LVOT and ascending aorta. These data are essential for determining procedural feasibility, vascular access route, and prosthesis selection tailored to the individual anatomy. CT also contributes to risk stratification for post-TAVR PVLs based on annular calcification and prosthesis-landing zone morphology [49,50]. In the post-implantation setting, the role of CT is more limited and mainly complementary to echocardiography. Thanks to its high spatial resolution and advanced multiplanar and 3D reconstruction capabilities, CT excels at identifying structural causes of PVLs such as: extensive or asymmetrical annular calcification, prosthesis malposition or migration, stent underexpansion [15].

The advantages and disadvantages of each imaging modality are summarized in Table 3.

## 4. Treatment of PVLs

The ReDo-TAVI registry, the leading data source on the efficacy and outcomes of PVL correction procedures, confirms the negative prognostic impact of moderate-to-severe PVL on survival. Data on mild PVL, however, remain conflicting. Some studies report a 21% mortality rate in moderate-to-severe PVL compared to 8% in mild cases.

The first and most effective treatment is prevention [51], through careful pre-procedural planning and proper selection of the bioprosthesis based on CT reconstruction.

### 4.1. Conservative Management

Before considering invasive treatment options, it is important to note that most PVLs are trivial or mild, and therefore management usually falls within an active surveillance strategy. As reported in a recent study [52], mild PVL developing after TAVR has no impact on mortality during a two-year follow-up period. Consistent with the findings of the previous PARTNER trial [3], PVL decreases progressively in approximately 31.5% of cases. The recent study further hypothesizes that this regression is primarily related to the gradual expansion of the valve “skirt”, driven by the increase in valve radius and by connective tissue proliferation—factors that contribute to the reduction in PVL in the months following implantation. Furthermore, the progressive improvement of transcatheter valve models has been accompanied by a marked reduction in this complication, as evidenced by the decline in PVL incidence from 68% in the PARTNER I trial to 28% in the PARTNER III trial. Altogether, these data support the view that, in cases of trivial or mild PVL, active surveillance with periodic echocardiographic or CT assessment often represents the most appropriate management strategy.

Once PVL at least moderate is detected, therapeutic strategies include:

- Bioprosthesis post-dilatation;
- Percutaneous PVL closure;
- TAVR-in-TAVR implantation.

### 4.2. Pre- and Post-Dilatation

Pre-dilatation was historically considered an essential step before TAVR to facilitate valve crossing and prosthesis delivery, ensure optimal expansion, and maintain hemodynamic stability during deployment. However, with procedural advancements, direct TAVR, performed without pre-implantation balloon aortic valvuloplasty, has emerged as a strategy to simplify the procedure and minimize valvuloplasty-related complications.

Available evidence supports the feasibility and safety of direct TAVI, with clinical outcomes comparable to those of procedures performed with pre-dilatation [53]. The decision to proceed with or without pre-dilatation should be guided by anatomical and procedural factors that may predispose to technical challenges, such as a small aortic annulus, difficulty in crossing the native valve, or severe valvular calcification potentially leading to suboptimal prosthesis expansion [53].

Post-dilatation is particularly useful in cases of heavily calcified aortic annuli, where under-expansion of the bioprosthesis can occur. When this is identified via angiography immediately post-implantation, prompt intervention is critical.

In a 2011 study [16], 79 patients underwent TAVR with CoreValve, and 21 of the 32 who developed moderate AR received post-dilatation. The procedure was successful (reduction in AR to below moderate) in 81% of these cases.

A more recent study [54] showed a 70% reduction in PVL regurgitation area following post-dilatation of balloon-expandable valves, without increasing neurological complications. However, other studies report a higher risk of cerebral embolism with this procedure. Known complications also include cusp damage (potentially leading to central AR), annular rupture (a potentially fatal event), and conduction disturbances requiring permanent pacemaker implantation (PPI). Therefore, although the use of post-dilatation has been accepted, concerns regarding its safety persist and further studies are warranted.

If post-dilatation is ineffective or contraindicated, percutaneous PVL closure should be considered.

### 4.3. Percutaneous PVL Closure

Closure using self-expanding double-disk nitinol occluder devices (e.g., Amplatzer^TM^ APVL3, Abbott, North Chicago, IL, USA) is a well-established approach. The technique involves retrograde femoral artery access, followed by PVL crossing with a guidewire and catheter delivery of the device.

In a cohort of 18 TAVR patients (self- and balloon-expandable valves), Waterbury et al. reported a 78% success rate (PVL reduction below moderate) following percutaneous closure [55]. Among the remaining 4 patients, 2 underwent post-dilatation and 2 underwent valve-in-valve procedures. Complication rates were low, but the study had limitations, including short follow-up and the off-label use of Amplatzer Vascular Plugs (AVPs), a family of braided—nitinol self-expandable devices, originally designed for peripheral artery embolization.

Careful pre-procedural planning using transthoracic/transesophageal echocardiography and CT is essential. The etiology and geometry of the PVL must be well understood, as serpiginous or eccentric leaks may complicate device deployment.

A 2023 systematic review by Umadzor et al., including 14 studies and 45 patients, reported a 94% success rate in PVL reduction (to below moderate) with low complication rates. At a mean follow-up of 21 ± 16 months, all patients maintained trace or mild PVL, regardless of the number of leaks treated [56,57].

### 4.4. TAVR-in-TAVR

Recent literature supports the use of TAVR-in-TAVR for correcting moderate-to-severe PVL, especially when the initial bioprosthesis is misaligned with the native annulus plane. A second valve may seal the leak by adding material and enhancing coaptation.

A study published in 2024 on 31 patients treated with balloon-expandable valves reported a 96% procedural success rate with low in-hospital complication rates, despite an elderly, high-risk population. At 2-year follow-up, 88% maintained mild or less PVL, and 70% showed NYHA class improvement, confirming a strong clinical benefit [58].

However, the effectiveness of this approach depends on PVL etiology. TAVR-in-TAVR is not effective when the leak is due to incomplete apposition of the first valve to the annulus. In such cases, percutaneous closure with an Amplatzer device remains the preferred alternative [55].

## 5. Conclusions

In conclusion, the issue of perivalvular leaks remains a highly relevant topic and continues to pose significant challenges in both diagnosis and management. Current evidence indicates that, while the increasing number of TAVR procedures has made PVL an area of growing interest, the continuous refinement of transcatheter valve designs is expected to progressively reduce the incidence of this complication in the future. An optimal approach begins with thorough preoperative planning, carefully tailored to each patient and focused on identifying individual risk factors predisposing to PVL. Equally crucial is an accurate diagnosis using the most appropriate imaging modalities, which serves as the foundation for selecting the best therapeutic strategy, whether conservative management or corrective intervention.

## Figures and Tables

**Table 1:** Main PVLs predictors.

Anatomical Predictors of PVLs
Heavy, extensive and asymmetrical calcifications
Elliptical annular shape (eccentricity index >0.25)
Extremely large or small annuli
BAV
Steep aorto-LVOT angle
Discrepancy between LVOT and annulus diameter
Self-expandable prosthesis
Incorrect prosthesis positioning (too high, too low, hypo expansion)
Prosthesis–annulus mismatch (cover index)
Significant PAD
Low BMI

PVLs: paravalvular leak; BAV: bicuspid aortic valve; LVOT: left ventricle outflow tract; PAD: peripheral artery disease; BMI: body mass index.

**Table 2:** Classification of prosthetic aortic valve regurgitation.

Three Grades Classification	None/Trace	Mild		Moderate		Severe
Five Grades Classification	None/Trace	Mild	Mild–Moderate	Moderate	Moderate–Severe	Severe
Qualitative and Semi-quantitative parameters						
Jet features						
Wide jet origin	Absent	Absent	Absent	Present	Present	Present
Multiple jets	Possible	Possible	Often present	Often present	Usually present	Usually present
Jet path visible along the stent	Absent	Absent	Possible	Often present	Usually present	Present
Proximal flow convergence visible	Absent	Absent	Absent	Possible	Often present	Often present
E/A ratio	<1.0	<1.0	<1.0	≥1.5	≥1.5	≥1.5
Vena contracta width (mm) (CD)	Not quantifiable	<2	2 to <4	4 to <5	5 to <6	≥6
Vena contracta area (mm^2^) (3D CD)	Not quantifiable	<5	5 to <10	10 to <20	20 to <30	≥30
Jet width at its origin (% LVOT diameter) (CD)	Narrow (<5)	Narrow (5 to <15)	Intermediate (15 to <30)	Intermediate (30 to <45)	Large (45 to <60)	Large (≥60)
Jet density (CW Doppler)	Incomplete or faint	Incomplete or faint	Variable	Dense	Dense	Dense
PHT (ms) (CW Doppler)	>500	>500	Variable (200 to <500)	Variable (200 to <500)	Variable (200 to <500)	<200
Diastolic flow reversal in proximal descending aorta (PW Doppler)	Absent	Absent or brief early diastolic	Intermediate	Intermediate	Holodiastolic with end-diastolic velocity 20 to <30 cm/s	Holodiastolic with end-diastolic velocity ≥30 cm/s.
Diastolic flow reversal in abdominal aorta (PW Doppler)	Absent	Absent	Absent	Absent	Absent	Present
Circumferential extent of PVR (%) (CD)	Not quantifiable	<5	5 to <10	10 to <20	20 to <30	≥30
**Quantitative parameters**						
Regurgitant volume (mL/beat)	<15	<15	15 to <30	30 to <45	45 to <60	≥60
Regurgitant orifice area (mm^2^)	<5	<5	5 to <10	10 to <20	20 to <30	≥30
Regurgitant fraction (%)	<15	<15	15 to <30	30 to <40	40 to <50	≥50
**CMR regurgitant fraction (%)**	<15	<15	15 to <30	30 to <40	40 to <50	≥50

Adapted from Pibarot et al. [34] and Zoghbi et al. [35]. CD = color Doppler; CW = continuous wave; LVOT = left ventricular outflow tract; PHT = pressure half-time; PVR = paravalvular regurgitation; PW = pulsed wave.

**Table 3:** Pros and Cons of different imaging technologies.

Echocardiography		CMR		Cardiac-CT	
Pros	Cons	Pros	Cons	Pros	Cons
Widespread use, feasible in the majority of settings	Inter-operator variability	Most accurate exam for quantification of regurgitant volume, regardless of number and localization of PVLs	High cost, low availability, need for expert radiologist and cardiologist, need of contrast media, contraindication in case of severe renal failure	Fundamental in pre-TAVR planning. Able to predict the risk of PVLs	No dynamic and quantitative assessment. Radiation exposure. Risk of contrast-induced acute kidney injury.
Good accuracy, especially for moderate and severe PLVs	Limited by heavy calcifications, prosthetic frame shadowing and poor acoustic windows.	Gold standard for ventricle volumes and function	Low spatial resolution and limited ability to define the mechanism of PVLs	High spatial resolution, useful for underline mechanism	Low utility in first diagnosis and patient follow-up
LV dimension and contractility evaluation	Low sensitivity for anterior PVLs with TTE and for posterior PVLs with TEE.	Feasible for all body types, operator-independent, and highly reproducible	Possible low patient tolerance, and artifacts due to motion or arrhythmias	Useful for planning interventional treatment of PVLs	
Estimation of haemodynamic impact	Precise quantification is challenging with multiple and very eccentric jets		Potential overestimation of RV by including coronary diastolic flow		
Practical for long term follow-up					

LV = left ventricle; RV = regurgitant volume.

## Data Availability

No new data were created or analyzed in this study. Data sharing is not applicable to this article.
